# Prostaglandin E_2_ Regulates AMPA Receptor Phosphorylation and Promotes Membrane Insertion in Preoptic Area Neurons and Glia during Sexual Differentiation

**DOI:** 10.1371/journal.pone.0018500

**Published:** 2011-04-07

**Authors:** Kathryn M. Lenz, Christopher L. Wright, Ryan C. Martin, Margaret M. McCarthy

**Affiliations:** 1 Department of Physiology and Program in Neuroscience, University of Maryland School of Medicine, Baltimore, Maryland, United States of America; 2 Department of Biology, Loyola University Maryland, Baltimore, Maryland, United States of America; University of Chicago, United States of America

## Abstract

Sexual differentiation of the rodent brain is dependent upon the organizing actions of the steroid hormone, estradiol. In the preoptic area, a brain region critical for the expression of adult reproductive behavior, there are twice as many dendritic spine synapses per unit length on newborn male neurons compared to female neurons and this sex difference correlates with the expression of adult male copulatory behavior. The sex difference in the POA is achieved via estradiol's upregulation of the membrane-derived lipid signaling molecule prostaglandin E_2_ (PGE_2_); PGE_2_ is necessary and sufficient to masculinize both dendritic spine density and adult sexual behavior in rats. We have previously shown that PGE_2_ activates EP_2_ and EP_4_ receptors which increases protein kinase A (PKA) activity and that masculinized dendritic spine density and sex behavior are both dependent upon PKA as well as activation of AMPA type glutamate receptors. In the current experiments, we build upon this signaling cascade by determining that PGE_2_ induces phosphorylation of the AMPA receptor subunit, GluR1, which leads to increased AMPA receptor insertion at the membrane. Treating female pups on the day of birth with PGE_2_ induced the phosphorylation of GluR1 at the PKA-sensitive site within 2 hours of treatment, an effect that was blocked by co-administration of the PKA/AKAP inhibitor, HT31 with PGE_2_. Brief treatment of mixed neuronal/glial POA cultures with PGE_2_ or the cAMP/PKA stimulator, forskolin, increased membrane associated GluR1 in both neurons and glia. We speculate that PGE_2_ induced increases in AMPA receptor associated with the membrane underlies our previously observed increase in dendritic spine density and is a critical component in the masculinization of rodent sex behavior.

## Introduction

Sexual differentiation of the rodent brain is achieved largely via the action of the steroid hormone, estradiol [1]. The preoptic area (POA), a brain region necessary for the expression of many reproductive behaviors including male copulation and maternal behavior, is highly sensitive to the organizing effects of estradiol. Astrocytes of the POA are sexually dimorphic, with males having longer and more numerous processes as a result of estradiol action during the neonatal period [2]. Additionally, POA neurons in males have over twice the number of dendritic spines as females due to a greater density of spines per unit length of dendrite, and neonatal estradiol exposure masculinizes the pattern of spines in females [3], [4]. Dendritic spines are the primary sites of excitatory synaptic input in the brain and frequently change in number and/or density in response to both internal and external variables associated with regulation of behavior. To wit, synaptic activity in the POA is necessary for the expression of male copulatory behavior in adulthood [5], and the masculine pattern of spines in the POA is similarly necessary for the expression of male copulatory behavior [4].

A substantial portion of the signal transduction pathway by which estradiol masculinizes the preoptic area during the neonatal critical period has been elucidated. The process begins with increased production of the lipid signaling molecule, prostaglandin E_2_ (PGE_2_) which is both necessary and sufficient to produce masculinized spine density in the POA during the perinatal sensitive period [2], [3]. Moreover, males treated neonatally with a prostaglandin synthesis blocker show feminized synaptic patterning and reduced mounting and ejaculation as adults, and females treated neonatally with PGE_2_ show masculinized spine density and increased male mounting behavior in adulthood [4]. The masculinizing effects of PGE_2_ are mediated via the EP_2_ and EP_4_ receptors [6]. The EP_2_ and EP_4_ receptors couple to protein kinase A (PKA), and PKA is necessary for the masculinization of POA spine density and male copulatory behavior [7]. Masculinization of both spine density in the POA as well as copulatory behavior are also dependent upon the activation of the AMPA-type glutamate receptor during the organizational period [3], [7]. Interestingly, PKA phosphorylates several AMPA receptor subunits, including GluR1 (or GluA1) [8], and this phosphorylation is promoted by the targeting of PKA to glutamate receptors by A kinase anchoring proteins (AKAPs) [9]. In other brain regions such as the hippocampus and cortex, GluR1 phosphorylation at the serine 845 residue results in increased AMPA receptor trafficking and insertion at dendritic spine synapses [8]. In the current studies, we sought to better connect PGE_2_-induced PKA activity with downstream effects on AMPA receptors by determining whether PGE_2_ leads to increased phosphorylation of GluR1 and AMPA receptor trafficking to the membrane, and whether these effects of PGE_2_ on GluR1 are in fact mediated by PKA.

## Methods

### Animals

All breeding and experimental procedures were approved by the Institutional Care and Use Committee at University of Maryland Baltimore and performed in accordance with national animal care and use guidelines. Adult Sprague Dawley rats (Harlan, Indianapolis, IN) were mated in our facility. Animals were maintained on a 12∶12 h light/dark cycle, with *ad libitum* access to food and water. Pregnant dams were allowed to deliver naturally. On the day of birth (postnatal day (PN) 0), pups were sexed and female pups treated within 6 hours of birth.

### In vivo manipulations

Bilateral intracerebroventricular (icv) injections were performed under cryoanesthesia. A 23 gauge 1 µl Hamilton syringe attached to a stereotaxic manipulator was placed 1 mm caudal to Bregma and 1 mm lateral to the midline, lowered 3.0 mm into the brain, and then backed out 1 mm. One µl of drug or vehicle was infused over 60 s, and then the procedure was repeated on the other hemisphere. The drug doses and timing of dosing specified below were all based on those shown previously to be effective in the neonatal POA [7].

### Microdissection of POA

Two hours following final drug treatment, pups were killed by rapid decapitation, their brains removed and placed in a Zivic Miller brain block, dorsal surface down. A 1 mm coronal section of the brain was taken using the rostral and caudal boundaries of the optic chiasm as landmarks. The anterior commissure served as the dorsal boundary of the POA. The isolated POA tissue was flash frozen on dry ice and stored at −80°C.

### Western blot

Tissue was homogenized in RIPA buffer containing 1% Igepal CA630, 0.25% deoxycholic acid, 1 mM EDTA, 154 mM NaCl, and 65 mM Trizma Base, with added protease and phosphatase inhibitors (1∶1000). All chemicals were obtained from Sigma unless otherwise specified. Protein supernatant was extracted after 20 minutes of centrifugation at 3000 rpm at 4°C, and total protein concentration determined via Bradford assay. Fifteen µg protein was loaded and electrophoresed on an 8–16% precast SDS polyacrylamide gel (Invitrogen) and transferred onto a single polyvinyl difluoride membrane (Bio-Rad) per experiment. Membranes were blocked for 60 min in 10% nonfat milk in 0.1% Tween in Tris-buffered saline (TTBS). Membranes were subsequently incubated with primary anti-sera in 5% milk in TTBS overnight at 4°C. Anti-sera were used at the following concentrations: pGluR1-s845 (Millipore, 1∶500); pGluR1-s831 (Upstate, 1∶1000); total GluR1 (Upstate, 1∶5000); GluR2 (Millipore 1∶1000); spinophilin (Millipore, 1∶1000). Membranes were rinsed, and appropriate HRP-conjugated secondary antibodies were applied in 5% milk in TTBS for 30 minutes. A Phototype chemilluminescence system (New England Biolabs) was used to detect the immunoblots by exposing the membrane to Hyperfilm ECL (GE Healthcare). Integrative grayscale pixel area densitometry captured with a CCD camera was quantified with NIH Image software. Ponceau staining was used as a loading control, and final immunoblot densitometry values for each lane expressed as a percentage of Ponceau staining for the same lane. Ponceau is our preferred loading control because it measures over 200 proteins, and is thus not sensitive to sex or hormonal manipulations, as are other common loading controls, such as GAPDH [10].

### POA primary neuronal culture

On PN0, the POA from female pups (*n* = 6–10) was microdissected as described as above, placed into 2 ml of HBSS and digested with 500 µl of 0.25% trypsin (Invitrogen) and 250 µl of 1% DNASE for 15 min at 37°C. The supernatant was removed, and cells were washed twice with HBSS. Cells were then gently triterated with a Pasteur pipette in cell culture media consisting of DMEM/F-12 (Invitrogen), 5% fetal bovine serum (Invitrogen), and 1% antibiotic/antimycotic (Invitrogen) until dissociated. Cell density and viability were determined on a hemacytometer using Trypan blue, and cells plated at 500,000 cells/slip in a 100 µl volume on 2.5 cm round poly-lysine treated glass coverslips in 3.5 cm Petri dishes. After allowing cells to seed for 2 hours, cultures were fed with 2 ml of cell culture medium, and allowed to acclimate and grow for 48 hours before treatment.

### Fluorescence immunohistochemistry

**Figure 1:**
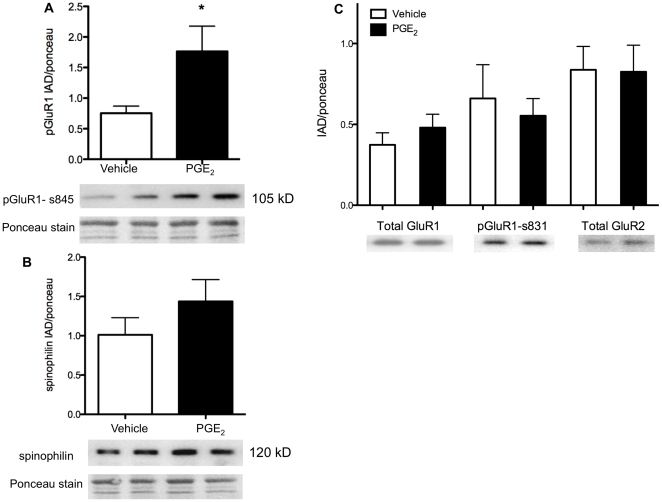
PGE_2_ and AMPA receptor subunit phosphorylation in the POA. Graphical representation and representative immunoblots for phosphorylated and total AMPA receptor subunit expression in the preoptic area following 2 hours of vehicle control or PGE_2_ treatment, corrected for loading with Ponceau staining. A) On the day of birth, PGE_2_ treatment significantly increased phosphorylation of GluR1 at serine 845, the PKA site (upper blot; band at 105 kDa) relative to vehicle-treated control pups. B) In the same animals there was no significant effect of short-term PGE_2_ treatment on spinophilin levels (upper blot; band at 120 kDa) relative to vehicle treated controls. At longer time points after PGE_2_ exposure (24–48 hrs), there is a robust and reliable increase in spinophilin levels [3]. The current results indicate that PGE_2_-induced phosphorylation of GluR1 precedes the up regulation of spinophilin. C) The effects of PGE_2_ treatment are amino acid residue specific as there was no effect on the expression of total GluR1 (left), GluR1 phosphorylation at serine 831 (PKC/CAMKII site; middle), or total GluR2 (right). * throughout denotes significantly different from vehicle-treated controls, *p*<0.05.

Cells were then fixed with 4% paraformaldehyde in PBS for 20 minutes, rinsed 3 times with PBS, and blocked for 1 hour with 5% bovine serum albumin (BSA) in PBS. Triton-X was omitted to prevent permeabilization and ensure only surface GluR1 labeling. Non-permeabilized cells were then incubated overnight at 4°C in antiserum to the N-terminus of GluR1 (Santa Cruz; goat 1∶200) in PBS +2.5% BSA, again omitting Triton-X. Coverslips were thoroughly rinsed in PBS, permeabilized with 0.4% Triton-X in PBS, and incubated for 2 hours with antiserum against microtubule associated protein 2 (MAP-2) as a neuronal marker (Sigma; mouse 1∶1000) in PBS +0.4% Triton-X +2.5% BSA. Coverslips were rinsed and incubated for 2 hours at RT in the dark in Alexafluor 594 donkey anti-mouse (1∶200) and Alexafluor 488 donkey anti-goat (1∶333) in PBS +0.4% Triton-X +2.5% BSA, rinsed again and mounted with Vectashield Hardset (Vector) onto gelatin treated slides. A subset of coverslips were co-incubated with GluR1 and MAP-2 without Triton-X; negligible MAP-2 staining in these cultures served as a positive control that the cells in the non-Triton-X condition remained non-permeabilized during GluR1 primary antibody incubation (Fig. 1C).

An Olympus BX51 microscope, QImaging Retiga SRV cooled CCD camera system and software, and an X-cite series 120Q light source were used for culture image acquisition. All images were obtained during a single imaging session at 20× magnification, using identical acquisition parameters, including camera exposure time, gain, gamma, offset, and fluorescence illumination intensity. Dual fluorescent images were obtained for 488 nm and 594 nm fluorophores using standard FITC and Texas Red filter sets, respectively. Experimenters were blind to experimental condition during image acquisition. Fields chosen for analysis contained at least one neuron with clearly identifiable neurites and at least one glial cell. Intensity of GluR1 expression was determined offline using Image J software. Cells co-labeled for MAP-2 and GluR1 were considered neurons, and analysis lines drawn around the soma and one randomly selected neurite per field to determine mean intensity of staining within the traced region. For every field in which a neuron was analyzed, a glial cell was also analyzed by the same outlining procedure. Glial cells were chosen based on the absence of MAP-2 staining and their characteristic morphology. Background staining intensity was also determined for each field analyzed, and the specific GluR1 staining of neurons and glia corrected by dividing raw intensity by the measured background.

### Data analysis

Data were analyzed using two-tailed *t*-tests or one-way analyses of variance (ANOVAs) coupled with Tukey's post-hoc tests when significant main effects were found.

### Experiment 1: Rapid effect of PGE_2_ on AMPA receptor phosphorylation and spinophilin expression

On PN0, female pups were either treated *icv* with 2.5 µg PGE_2_ (Sigma) in 0.9% saline (*n* = 10) or saline vehicle (*n* = 9), allowed to recover from cryoanesthesia under a heat lamp, and returned to the dam until euthanasia 2 hours following injection. POA tissue was frozen at 80°C until western blots were performed for phosphorylated AMPA receptor subunits or the dendritic spine marker, spinophilin, which has previously been shown to be up regulated by PGE_2_ [4], [7]. Additional samples were generated in the same manner as above to assay for total GluR1 and GluR2 protein levels (vehicle *n* = 4; PGE_2_ *n* = 5).

### Experiment 2: PKA dependence of PGE_2_ effects on phosphorylation of GluR1

On PN0, female pups were first treated *icv* with 5.5 µg of HT31 or the corresponding control peptide (CP) (Promega). HT31 inhibits the interaction between PKA and the A Kinase Anchoring Protein (AKAP), which has been shown to be crucial for AMPA receptor phosphorylation [9]. The corresponding control peptide has 2 amino acid substitutes that prevent its ability to inhibit PKA/AKAP interaction. Between 45–60 minutes following treatment with HT31 or CP, pups were subsequently treated *icv* with either 2.5 µg PGE_2_ (Sigma) in 0.9% saline or saline vehicle, allowed to recover from cryoanesthesia under a heat lamp, and returned to the dam until euthanasia 2 hours following injection. Four groups resulted: CP + vehicle (*n* = 6); CP + PGE_2_ (*n* = 6); HT31 + vehicle (*n* = 8); and HT31 + PGE_2_ (*n* = 7).

### Experiment 3: Effects of PGE_2_ and PKA on surface GluR1 expression in POA primary cell cultures

On DIV 2, female POA cultures were treated with either 20 nM PGE_2_ in 0.1 M PBS, PBS vehicle, the cAMP/PKA stimulator forskolin in DMSO (10 µM) or DMSO vehicle for 30 minutes and processed for fluorescence immunohistochemistry against GluR1 membrane expression. This dose of forskolin has previously been used by our laboratory to produce significant increases in dendritic spines in cultured POA neurons [7]. Cultures were co-labeled with MAP-2 to identify GluR1-positive neurons from GluR1-positive glia, which are also present in the POA. Intensity of membrane GluR1 staining was quantified as specified above. A total of 6–7 coverslips per treatment group were imaged, with 8–10 neurons and glia analyzed per coverslip. Mean neuronal and glial fluorescence intensity over background fluorescence was then determined for each coverslip and these means compared across groups using t-tests. All data collection and imaging was performed blind to condition.

## Results

### Experiment 1

PGE_2_ treated female pups had a significant increase in GluR1 phosphorylation at serine 845 (the PKA site) in the POA relative to vehicle treated female pups (*t* (10) = 2.35, p<0.05; *df* Welsh-corrected for unequal variances; Fig. 1A). Two hours following treatment with PGE_2_ or vehicle, there was no difference in spinophilin levels in the POA (*t* (17) = 1.19, *ns*; Fig. 1B).

Vehicle and PGE_2_ treated samples were also assayed to determine levels of total GluR1, total GluR2, and GluR1 phosphorylation at the serine 831 (PKC/CAMKII) site. Two hours of PGE_2_ treatment had no effect on total GluR1 expression (*t* (7) = 0.93, *ns*), GluR1 phosphorylation at serine 831 (*t* (7) = 0.49, *ns*) or total GluR2 expression (*t* (7) = 0.05, *ns*; Fig. 1C).

### Experiment 2

**Figure 2:**
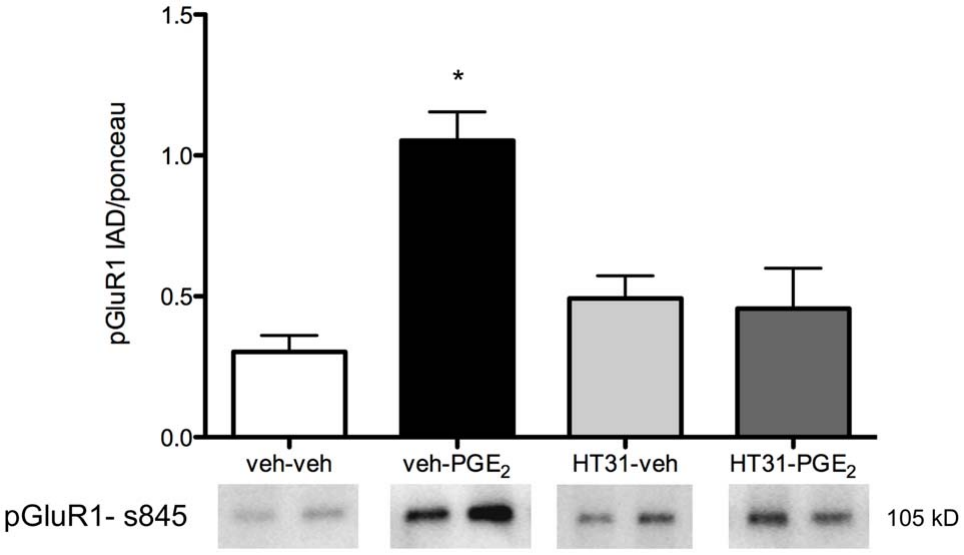
PKA dependence of PGE_2_ effects on GluR1 phosphorylation in the POA. After two hours of PGE_2_ treatment there was again significantly increased pGluR1 (s845) relative to vehicle treated controls (veh-PGE_2_). Pretreatment of pups with the PKA/AKAP inhibitor, HT31 abolished this PGE_2_-dependent up regulation (HT31-PGE_2_). Neither HT31 + vehicle or HT31 + PGE_2_ treatments produced significant effects on GluR1 phosphorylation in the POA. * denotes significantly different from vehicle-treated controls, *p*<0.05.

PGE_2_ once again significantly increased GluR1 phosphorylation at the PKA site, serine 845 (*F* (3, 23) = 9.19, *p*<0.001), but co-administration of the PKA/AKAP inhibitor HT31 with PGE_2_ resulted in pGluR1 levels that were not significantly different from animals treated with vehicle or control peptide (Fig. 2).

### Experiment 3

**Figure 3:**
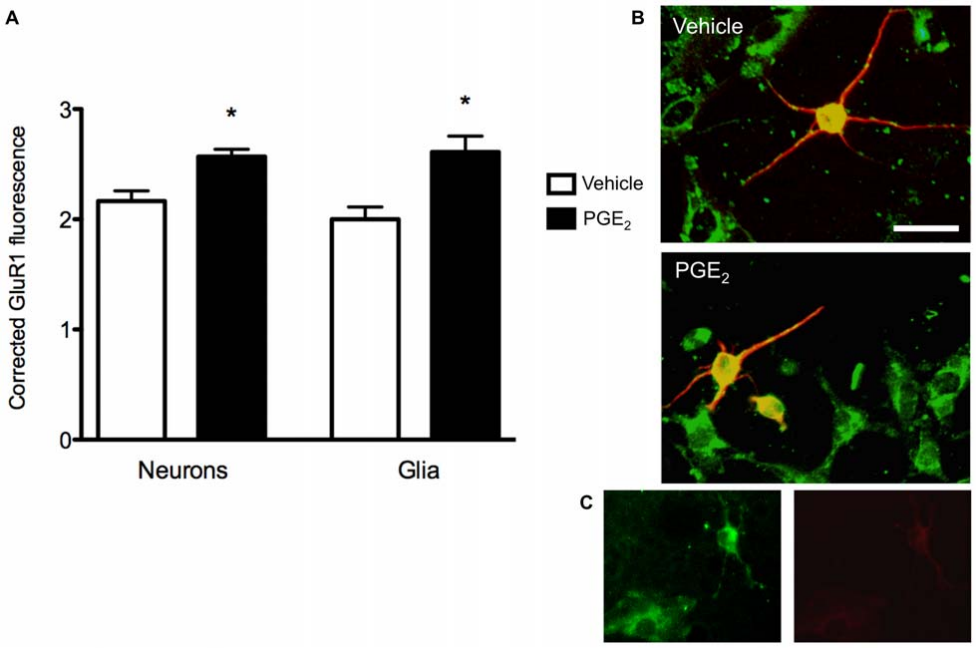
Effects of PGE_2_ on membrane association of GluR1 in dissociated POA cell cultures. A) PGE_2_ treatment (20 nM) significantly increased the surface association of GluR1 on both neurons and glia relative to vehicle-treated controls. The intensity of fluorescence on neurons and glia was corrected for background staining throughout. * denotes significantly different from vehicle-treated controls, *p*<0.05. B) Representative composite fluorescence digital photomicrograph of cells from a vehicle treated culture (top) and a PGE_2_ treated culture (bottom) at 20× magnification. Scale bar  = 25 µm. Green labeling is for GluR1 and red for MAP-2 as a neuronal marker. Co-labeled cells (yellow) are neurons, and green cells (e.g., those without MAP-2 staining) are putative glial cells. PGE_2_ treated cultures show increased intensity of membrane-associated GluR1 staining (green) in both neurons and glia relative to vehicle treated cultures. C) Control cultures which were not permeablized before immunohistochemistry for GluR1 (extracellular domain specific) and MAP-2 show ample GluR1 staining in neurons and glia (left) and negligible background staining for MAP-2 (right).

**Figure 4:**
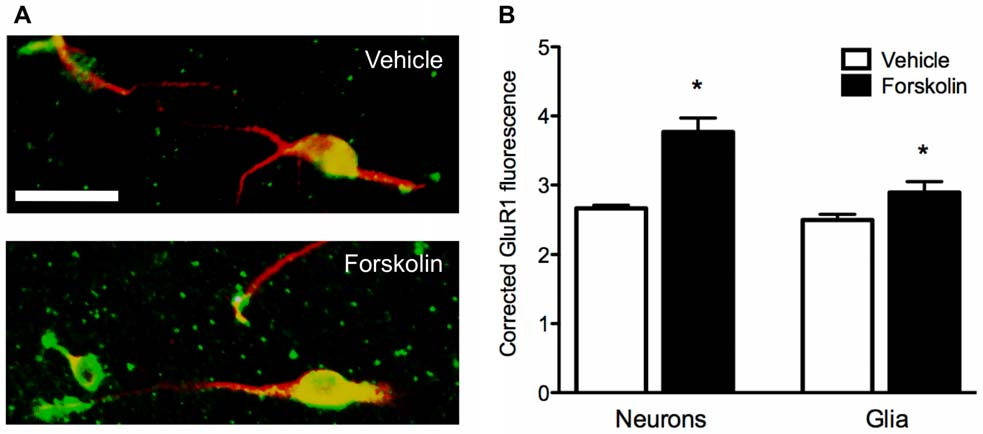
Effects of forskolin on membrane association of GluR1 in dissociated POA cell cultures. A) Representative composite fluorescence digital photomicrograph of cells from a vehicle treated culture (top) and a forskolin treated culture (bottom) at 20× magnification. Scale bar  = 20 µm. Green labeling is for GluR1 and red for MAP-2 as a neuronal marker. Co-labeled cells (yellow) are neurons, and green cells (e.g., those without MAP-2 staining) are putative glial cells. B) Forskolin treatment (10 µM) significantly increased the surface association of GluR1 on both neurons and glia relative to vehicle-treated controls. The intensity of fluorescence on neurons and glia was corrected for background staining throughout. * denotes significantly different from vehicle-treated controls, *p*<0.05.

All POA cultures showed substantial neuronal and glial surface GluR1 expression (Figs. 3 and 4). However, cultured POA neurons treated with PGE_2_ for 30 minutes showed an acute increase in surface staining for GluR1 relative to saline treated cultures (*t* (11) = 3.63, *p*<0.01; Fig. 3). Glia from POA cultures also showed up regulated GluR1 surface staining in response to treatment with PGE_2_ (*t* (11) = 3.25, *p*<0.01; Fig. 3). Stimulating PKA also influenced GluR1 membrane expression, with forskolin upregulating surface GluR1 levels in both neurons (*t* (5) = 5.322, *p*<0.005; *df* Welsh-corrected for unequal variances) and glia (*t* (10) = 2.25, *p*<0.05) relative to DMSO-treated control cultures (Fig. 4). No differences in average background staining were found between groups in the PGE_2_ experiment (vehicle  = 6.26±0.37; PGE_2_  = 5.62±0.26; *t* (11) = 1.47, *ns*) or the forskolin experiment (vehicle  = 9.19±0.60; forskolin  = 8.22±0.78; *t* (10) = 0.98, *ns*).

## Discussion

Previous research from our laboratory has shown that prostaglandin E_2_ is necessary and sufficient to masculinize dendritic spine density in the developing preoptic area, and that the neonatal establishment of this masculine morphology strongly correlates with the expression of adult male sex behavior. PGE_2_ acts via its EP_2_ and EP_4_ receptors to recruit PKA, and ultimately results in increased signaling via the AMPA-type glutamate receptor; Both PKA activity and AMPA receptor activation are also necessary for the masculinization of sex behavior [7]. What has been missing is an understanding of how PGE_2_-induced PKA activity impacts on AMPA receptor functioning. In the current studies, we have filled this gap by showing that PGE_2_ rapidly up regulates phosphorylation of the GluR1 subunit of the AMPA receptor by PKA, and also increases insertion of GluR1 into the membrane, both of which likely contribute to the formation and stabilization of nascent dendritic spines. In addition, we have shown that PKA activation is sufficient to increase GluR1 membrane levels in POA neurons, in keeping with similar effects of PKA in brain regions such as the hippocampus and cortex.

PGE_2_ induced increases in dendritic spines on developing POA neurons occurs over a 24–48 period. Here we show that treatment of females on the day of birth with PGE_2_ produces an increase in phosphorylated GluR1 within 2 hours, and this precedes the prostaglandin-driven upregulation of spinophilin protein. In light of our previous studies showing that both PKA signaling as well as AMPA receptor activation and the resultant masculinization of dendritic spines in the POA are necessary for the expression of male sex behavior, the current results suggest that one substrate of PKA activity is the GluR1 subunit of the AMPA receptor, and that this GluR1 phosphorylation contributes to dendritic spine stabilization and postsynaptic AMPA receptor signaling. This model is also consistent with previous findings from our laboratory showing that AMPA receptor antagonism in the developing POA does not disrupt already masculinized spinophilin levels in males (e.g., spine maintenance), but does prevent the masculinization of spinophilin levels in PGE_2_-treated females (e.g., spine induction) [3].

Several AMPA receptor subunits, including GluR1, GluR2, and GluR4, are dynamically regulated by protein kinases A and C, calcium-calmodulin dependent kinase 2, and receptor tyrosine kinases (reviewed in [11]). Subunit phosphorylation has been implicated in a variety of biological processes, including receptor internalization or externalization, which then can lead to morphological changes (e.g., spine formation or stabilization, synapse elimination) as well as physiological changes (e.g., long term potentiation, long term depression, altered AMPA current size). Given that PKA activity is necessary for the PGE_2_-induced masculinization of the POA and copulatory behavior [7], we chose to focus on PKA-mediated phosphorylation at the serine 845 residue on GluR1. Indeed, we found that disrupting PKA targeting to macromolecular signaling complexes with the AKAP inhibitor, HT31, prevented PGE_2_ from up regulating GluR1 phosphorylation at serine 845 in the neonatal POA.

Phosphorylation of serine 845 phosphorylation of GluR1 has been implicated in the potentiation of the post-synaptic AMPA current [12] as well in inserting AMPA receptors into the cell surface [13] or more specifically, the synapse [8]. This role of GluR1 serine 845 phosphorylation in AMPA receptor insertion is consistent with our observed effect of PGE_2_ on GluR1 membrane expression in cultured POA cells. We found that PGE_2_ up regulated serine 845 phosphorylation in the POA while not affecting the serine 831 residue on GluR1, which is a favored phosphorylation site for PKC and CAMKII activity [12], suggesting a specific effect of PGE_2_ on PKA as opposed to a nonspecific increase in kinase activity. Additionally, we found no effect of PGE_2_ treatment on total GluR1 expression in the POA, therefore the observed effects on pGluR1 s845 were not attributable to overall increases in GluR1, but instead site-specific phosphorylation of serine 845.

In addition to GluR1, PKA phosphorylation of GluR4 is also associated with AMPA receptor insertion into synapses [8]. Interestingly, GluR4 is preferentially expressed in the neonatal brain [14], potentially implicating it in circuit and synapse development. Therefore, though the current results reveal PGE_2_ induced phosphorylation of GluR1 and increased membrane GluR1 expression, we cannot rule out that GluR4 also contributes to sexual differentiation of spine density in the POA. Similarly, while the current experiments focused on the role of prostaglandin-induced upregulation of PKA activity on the AMPA receptor, previous research from our laboratory has also shown that PKA regulation of metabotropic glutamate receptors plays a role in the induction of dendritic spines in the POA and the organization of adult male sex behavior [7].

In addition to increased trafficking of GluR1 to neuronal membranes, we also observed increased surface expression of GluR1 on glia after PGE_2_ treatment. Both neurons and glia in the POA are sexually differentiated by estradiol [2], [3], but the precise contribution of each cell type to masculinized sex behavior has yet to be determined. This will require determining which cell type is sexually differentiated first, and whether the differentiation of one cell type is dependent upon the differentiation of the other. The fact that we find increased membrane GluR1 following PGE_2_ on both cell types suggests that glutamatergic signaling in both neurons and glia may be relevant for the organization of sex differences in the POA and adult male sex behavior. Cross talk between neurons and glia is important for spinogenesis [15], [16], and prostaglandins have been implicated in this communication between neurons and glia [17]. Prostaglandins induce glial release of glutamate [18]; glutamate can then act on neurons to regulate spine morphogenesis and synaptic plasticity. Glutamate itself can also act on glial AMPA receptors [18], [19], and can further elicit glial release of glutamate [18], [20] as well as release of prostaglandins [18]. Therefore, neurons and glia in the POA likely engage in a type of mutual positive feedback involving glutamate, and this continued communication may be crucial for the sexual differentiation of the POA.

In cortex and hippocampus PGE_2_ increases excitability and contributes to the induction of LTP [21], [22], [23], [24] and the effects of PGE_2_ on excitability, firing rate and EPSP amplitude in the hippocampus are dependent upon PKA [21]. In the spinal cord, PKA anchoring by AKAP is necessary for PGE_2_ induced changes in post-synaptic excitability to occur [25]; our current finding that PGE_2_ effects on AMPA receptor dynamics are dependent upon PKA/AKAP suggests this may also be the case in the preoptic area. Over expression of AKAP in dissociated hippocampal cultures also increases spine-like protrusions as well as AMPA receptor localization post-synaptically [26], which is consistent with our findings previously and in the current experiments that prostaglandin-induced PKA/AKAP signaling is relevant for AMPA receptor insertion and spinogenesis. Therefore, our studies contribute to a greater understanding of prostaglandin-dependent regulation of glutamatergic signaling, as well as implicating the PKA/AKAP postsynaptic signaling complex as a regulator of AMPA receptor dynamics, the formation and stabilization of dendritic spines, and eventually a sexually differentiated brain and masculine behavioral phenotype.

Overall, the current experiments add to our understanding of the mechanisms through which estradiol during the neonatal critical period induces sexual differentiation of the preoptic area and masculinizes sex behavior. Estradiol acts to upregulate COX-1 and COX-2, the synthesizing enzymes for prostaglandin E_2_ [4]. Elevated PGE_2_ activates PKA via its EP_2_ and EP_4_ receptors [6], and PKA phosphorylates the glutamate receptor subunit, GluR1, which in turn corresponds to increased GluR1 trafficking into the cell membrane of both neurons and glia. Subsequent activation of glutamate receptors leads to increased spinophilin and the induction of dendritic spine synapses in the POA, which are necessary for the expression of male sex behavior in adulthood.
